# Application of Zinc and Iron-Based Fertilizers Improves the Growth Attributes, Productivity, and Grain Quality of Two Wheat (*Triticum aestivum*) Cultivars

**DOI:** 10.3389/fnut.2021.779595

**Published:** 2021-12-13

**Authors:** Muhammad Bilal Hafeez, Yasir Ramzan, Shahbaz Khan, Danish Ibrar, Saqib Bashir, Noreen Zahra, Nabila Rashid, Majid Nadeem, Saleem ur Rahman, Hira Shair, Javed Ahmad, Makhdoom Hussain, Sohail Irshad, Abdulrahman Al-Hashimi, Alanoud Alfagham, Zeng-Hui Diao

**Affiliations:** ^1^Wheat Research Institute, Ayub Agricultural Research Institute, Faisalabad, Pakistan; ^2^National Agricultural Research Centre, Islamabad, Pakistan; ^3^Department of Plant and Soil Sciences, Oklahoma State University, Ardmore, OK, United States; ^4^Department of Soil and Environmental Science, Ghazi University, Dera Ghazi Khan, Pakistan; ^5^Department of Botany, University of Agriculture, Faisalabad, Pakistan; ^6^Department of Agronomy, Muhammad Nawaz Shareef University of Agriculture, Multan, Pakistan; ^7^Department of Botany and Microbiology, College of Science, King Saud University, Riyadh, Saudi Arabia; ^8^Guangdong Provincial Engineering and Technology Research Center for Agricultural Land Pollution Prevention and Control, Zhongkai University of Agriculture and Engineering, Guangzhou, China

**Keywords:** Anaj-17, biofortification, grain quality, micronutrients, wheat growth, Zincol-16

## Abstract

Field-based experiments were conducted during wheat cultivation seasons of 2017–2018 and 2018–2019 to minimize the impact of hidden hunger (micronutrient deficiencies) through agronomic biofortification of two wheat cultivars with zinc and iron. Two spring-planted bread wheat cultivars: Zincol-16 (Zn-efficient) and Anaj-17 (Zn-inefficient with high-yield potential) were treated with either zinc (10 kg/ha), iron (12 kg/ha), or their combination to study their effect on some growth attributes (plant height, tillers, and spike length, etc.,), productivity, and quality. No application of zinc and iron or their combinations served as the control. Maximum Zn and Fe contents of grains were improved by sole application of Zn and Fe, respectively. A higher concentration of Ca in grains was observed by the combined application of Zn and Fe. Starch contents were found maximum by sole application of Fe. Sole or combined application of Zn and Fe reduced wet gluten contents. Maximum proteins were recorded in Anaj-17 under control treatments. Zincol-16 produced maximum ionic concentration, starch contents, and wet gluten as compared to Anaj-17. Yield and growth attributes were also significantly (*p* < 0.05) improved by combined application as compared to the sole application of Zn or Fe. The combined application also produced the highest biological and grain yield with a maximum harvest index. Cultivar Anaj-17 was found more responsive regarding growth and yield attributes comparatively. The findings of the present study showed that the combined application of Zn and Fe produced good quality grains (more Zn, Fe, Ca, starch, and less gluten concentrations) with a maximum productivity of bread wheat cultivars.

## Introduction

Globally, the deficiencies of dietary micronutrients are widespread and pose a major health concern for more than 2 billion people (1–3). After the green revolution, scientist's basic concern is to increase productivity rather than the quality of edible crop parts (4). That is the reason, malnutrition is one of the foremost tasks for agricultural scientists (5). Worldwide, zinc (Zn) and iron (Fe) deficiencies are the most widespread micronutrient disorder. Zn deficiency causes gastrointestinal problems (6), altered reproductive biology, impairments of physical growth (7), DNA damage and cancer development (8), diabetes mellitus, hormone imbalance, respiration issues and high blood pressure, and affects multiple aspects of the immune system. Fe deficiency causes anemia and pregnancy issues (9), tiredness and a poor immunity level, reduced work capacity and intellectual performance, less cognitive development, growth, and reproductive performance (10).

Monotonous and excessive use of wheat-based products has rapidly increased malnutrition. Among cereals, wheat is one of the staple crops, which is being consumed as a staple food for 1.2 billion of the world population (11). Globally, wheat was cultivated in the year 2019 about an area of 214.7 million hectares (M ha) and was the second-highest cereal crop with the production of 749 million tons (MT) after maize (12). In Pakistan (2018), wheat was grown on an area of 8.79 M ha with a production of 25.076 MT that was surplus than country demand (13). People in rural regions fulfilled their 70% daily calories through the wheat, and 60% of the population consumed wheat as a basic dietary food (14). The requirement for wheat to feed the escalating world population is expected to increase up to 40% by 2050 to meet food security (15, 16). According to a survey (17), 25–30% of soil is calcareous and Fe deficient. Around the world, it is estimated that about 50% of soils, under wheat cultivation, are Zn deficient (18). Zn and Fe deficiency is more common in Pakistani soil that has high pH, free CaCO_3_, and HCO^3−^, which inhibit the accessibility of Fe and Zn to the plants (16, 19).

Micronutrient-deficient soils are increasing due to the frequent growth of higher-yielding crops and intensive use of fertilizer, i.e., nitrogen, potassium, and phosphorus (20). In plants, Zn is a structural constituent/ activator of many enzymes involved in protein synthesis, regulation of auxin synthesis, carbohydrate metabolism, and membrane integrity (21, 22) and plays an important role in chlorophyll formation, pollen development, and fertilization (23), essential for the regulation of the gene expression needed for the tolerance of abiotic stresses in plants. Under acute zinc deficiency, visible symptoms include chlorosis of leaves, stunted growth, small leaves, and spikelet sterility (24, 25). In plants, Fe plays a vital role in chlorophyll synthesis as it is a component of cytochromes and electron transport (26). Its deficiency decreases the activity of various enzymes such as catalase and peroxidase that contain porphyrin as a prosthetic group (27). Fe chlorosis is also induced by HCO^−3^ that impairs the mechanism of Fe uptake (28). To mitigate the nutrient imbalance, plants use various mechanisms to reduce water loss while maximizing water uptake, including a reduction in the leaf area and osmotic adjustment through the application of liquid seaweed extract, organic compounds, and minerals elements (29–31).

Among various fortification techniques, agronomic biofortification is the most cost-effective, rapid, and sustainable strategy to improve the contents of micronutrients in wheat grains to alleviate the widespread Zn and Fe deficiencies in humans (32). Zinc sulfate (ZnSO_4_) and iron sulfate (FeSO_4_) are the most widely applied inorganic fertilizers as sources of Zn and Fe, respectively, due to their high solubility and low cost (33, 34). There is convincing proof about Zn and Fe fertilizer's effectiveness in improving their wheat grain concentrations and economic yield in Zn- and Fe-deficient regions (5, 35). The application of ZnSO_4_ and FeSO_4_ is reported as efficient in enhancing the quality of wheat grains (1, 34, 36). Several studies have demonstrated that soil supplementation of micronutrients showed good behavior in increasing their contents in wheat grain (5, 37–39). Exogenous applications of Zn and Fe may be useful to improve the quality of wheat grain with high production. After considering the above notation, a field-based trial was performed to reduce the impact of hidden hunger by assessing the following objective: (i) to compare the yield and physiological response of Zincol-16 (Zn-efficient) and Anaj-17 (Zn inefficient and high yielding) under soil-applied Zn and Fe, (ii) to explore the influence of sole and/or combined application of Zn and Fe on grain quality and yield attributes of wheat cultivars.

## Materials and Methods

### Experimental Field Location and Soil Specification

**Figure 1:**
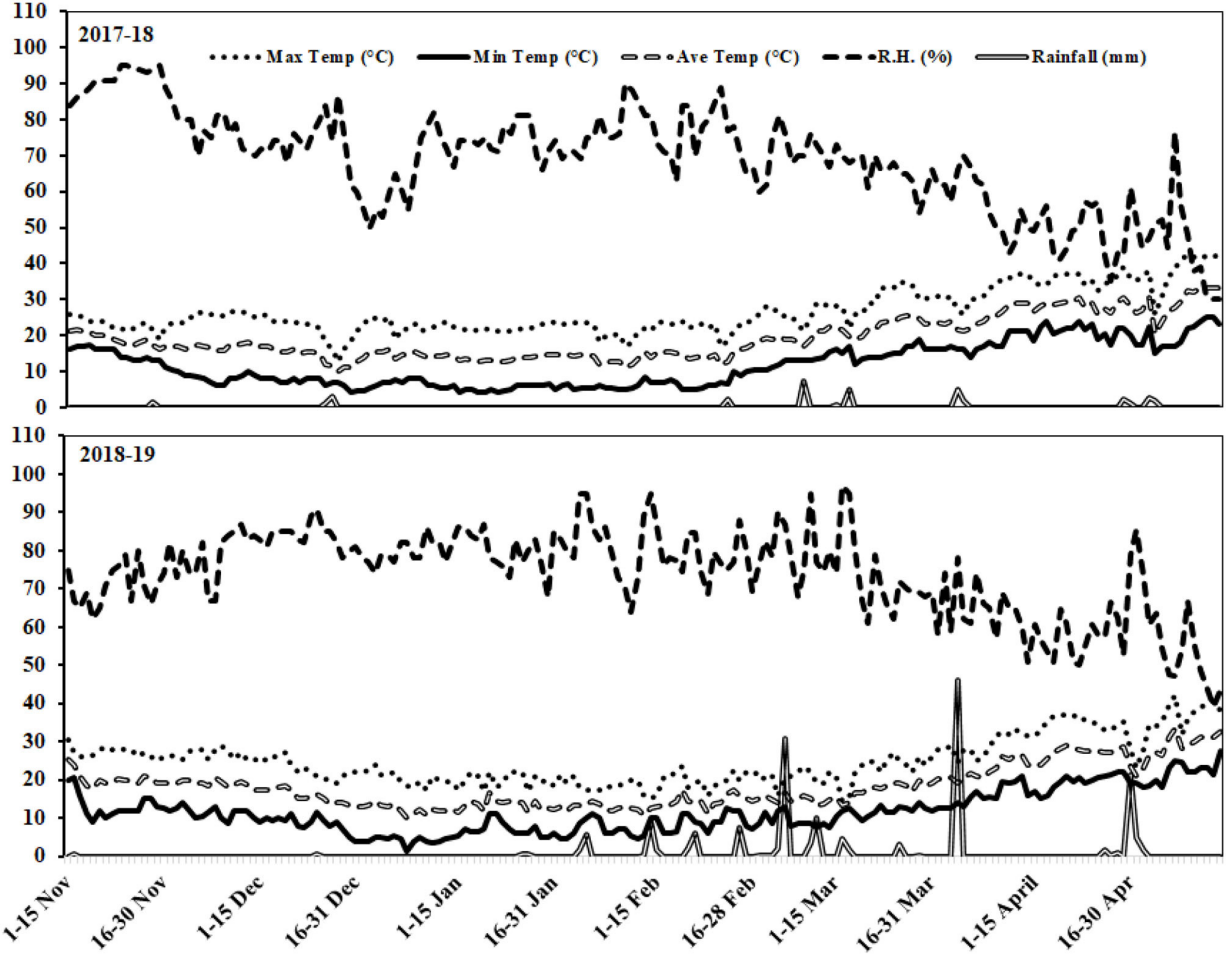
Weather data of the experimental station during 2017–2018 and 2018–2019 growing seasons.

A 2-year field trial was conducted at Farm Area of Wheat Research Institute (WRI), Faisalabad-Pakistan with longitudes of 73°74 East and latitude of 30°31.5 North with an elevation of 184 m (604 ft.) above the sea level. Faisalabad falls under a semiarid climatic zone due to high evapotranspiration with a mean annual rainfall of about 200 mm. Soil samples from different sites of the study area at 0-cm to 30-cm depth were collected after primary land preparation and prior to conducting the experiment. Estefan et al. (40) protocols were followed for analysis of soil physicochemical properties as presented in Table 1. Air-dry soil of 50 g was taken in a 100-ml glass beaker, and 50 ml of deionized water was added and shacked well for mixing. The suspension was allowed to stand for 30 min, and the working sample was prepared. The weather data, including maximum temperature, minimum temperature, average temperature, relative humidity, and rainfall, are presented in Figure 1.

**Table 1:** Physical and chemical analysis of soil of the field trial site.

**Characteristics**	**Units**	**Value (2017–18)**		**Value (2018–19)**	
**Soil depth**	**(cm)**	**0–15**	**15–30**	**0–15**	**15–30**
Texture	(Class)	Sandy clay loam	Sandy clay loam	Sandy clay loam	Sandy clay loam
pH		7.5	7.7	7.6	7.5
EC	(dS m^−1^)	2.31	2.36	2.25	2.33
Organic matter	(%)	0.72	0.67	0.73	0.71
Total nitrogen	(%)	0.041	0.038	0.039	0.035
Available P (Olson)	(mg kg^−1^)	4.7	4.4	4.9	5.1
Extractable K (NH_4_OAC)	(mg kg^−1^)	300	320	300	330
DTPA Zn	(mg kg^−1^)	0.51	0.47	0.52	0.44
DTPA Fe	(mg kg^−1^)	2.62	2.41	2.75	2.66

### Experimental Design and Treatments

Randomized complete block design with factorial (two cultivars and four treatments) arrangement was selected with three replications per treatment. The net plot was 5 × 2.25 m with a row spacing of 22.5 cm. Each experimental unit consists of 10 rows that were 5-m long. Zinc sulfate (ZnSO_4_) and iron sulfate (FeSO_4_) were used as the sources of zinc and iron, respectively. The given below treatment plan was applied to study the above-discussed objectives;

- Control (no soil application of zinc or iron).
- Sole application of zinc at 10 kg ha^−1^.
- Sole application of iron at 12 kg ha^−1^.
- Combined application of zinc and iron at 10 and 12 kg ha^−1^, respectively.

All the treatments were applied before sowing the crop. Zinc sulfate and iron sulfate were manually spread in the experimental field with recommended fertilizer doses.

### Cultivars Used

Two major spring-planted wheat cultivars (Zincol-16 and Anaj-17) were used for the study. Cultivar Zincol-16 is claimed as zinc efficient with low-yield potential, while Anaj-17 is considered zinc inefficient with high-yield potential. Seeds of both cultivars were collected from the gene bank of WRI. The cultivars are being widely grown in wheat-cultivated areas of this region of the world. A recommended seed rate of each cultivar at 100 kg ha^−1^ was used.

### Crop Cultivation and Management Practices

After harvesting the previous crop of rice from the field, a deep plow was used to break the hardpan, and stubbles were incorporated into the soil. Before sowing of seeds, seedbed was prepared by using a cultivator two times with the same number of plankings. Seeds of respective cultivars were sown in a well-prepared fine seedbed with the help of a Norwegian planter. Moreover, K, N, and P fertilization were applied at rates of 60 kg ha^−1^, 114 kg ha^−1^, and 120 kg ha^−1^, respectively. Urea (46% N), diammonium phosphate (18% N and 46% P_2_O_5_), and murate of potash (60% K_2_O) fertilizers were used as a source of primary nutrients. At the time of sowing, a complete dose of K and P with 1/3 of N was used as a basal dose. Remaining N was supplied with first and second irrigation with an equal split. Four-time irrigation was applied throughout the season of the wheat crop. Necessary plant protection measures were adopted to keep crops free of pests, weeds, insects, and diseases. All other agronomic practices were kept uniform throughout the course of experimentation. The crop was harvested manually after 160 days of sowing, left in the field for sun drying for a week. After sun-dried, spikes were threshed manually.

### Measurement of Quality Parameters

Mineral ions—Zn, Fe, Cu, Mg, and Ca—accumulation was determined in the grains of wheat cultivars (Zincol-16 and Anaj-17) that were collected from each experimental unit during both growing seasons. After threshing the spikes, the grain samples were carefully washed three times with deionized water with each 30 s and were dried at 65°C in an electric oven, typically ranging from ambient to 300°C. Mineral contents, such as Ca, Mg, Cu, Zn, and Fe, in the grains were recorded according to the wet digestion method described by Rashid (41). Atomic absorption spectrum (Skemadzu 7,000) was used to analyze the mineral contents. Starch contents in the wheat grains were determined by following the protocol developed by Edwards (42). Amylose and amylopectin contents were measured through a fraction of 100 mg from each sample. Bradford technique (43) was used to determine the total soluble protein. To extract protein, 0.1 g of grain was grounded using a cooled phosphate buffer (pH 7.8) placed in an ice bath. The homogenate was centrifuged at 15,000 rpm for 5 min at 4°C. The supernatant was used for protein determination.

### Measurement of Growth and Yield Parameters

At harvesting, the number of tillers (m^−2^) was counted. Plant height and spike length were measured accurately with the help of a meter rod. The number of spikelets per spike and the grain number per spike were calculated manually than averaged. At the fully mature stage, an area of 1 m^2^ was harvested for the measurement of biological yield. Grain yield was measured by obtaining grains from each experimental unit after threshing. Additionally, 1,000-grain weight was recorded by using weight balance. Harvest index was also calculated with the following formula: grain yield/biological yield × 100.

### Statistical Analysis

Recorded data were analyzed and evaluated statistically using a statistical package (Statistix 8.1). Comparison among treatments was made by a two-way ANOVA technique at a CI of 95%. Various letters (a, b, c, etc.) were used to portray the significant difference among treatments *via* LSD as a *post-hoc* test. Microsoft Excel was used for calculation and graphical presentation. Mendeley Desktop (1.19.1) was used for citation and bibliography. Pearson correlation was drawn among different response variables.

## Results

### Ions Concentrations

**Figure 2:**
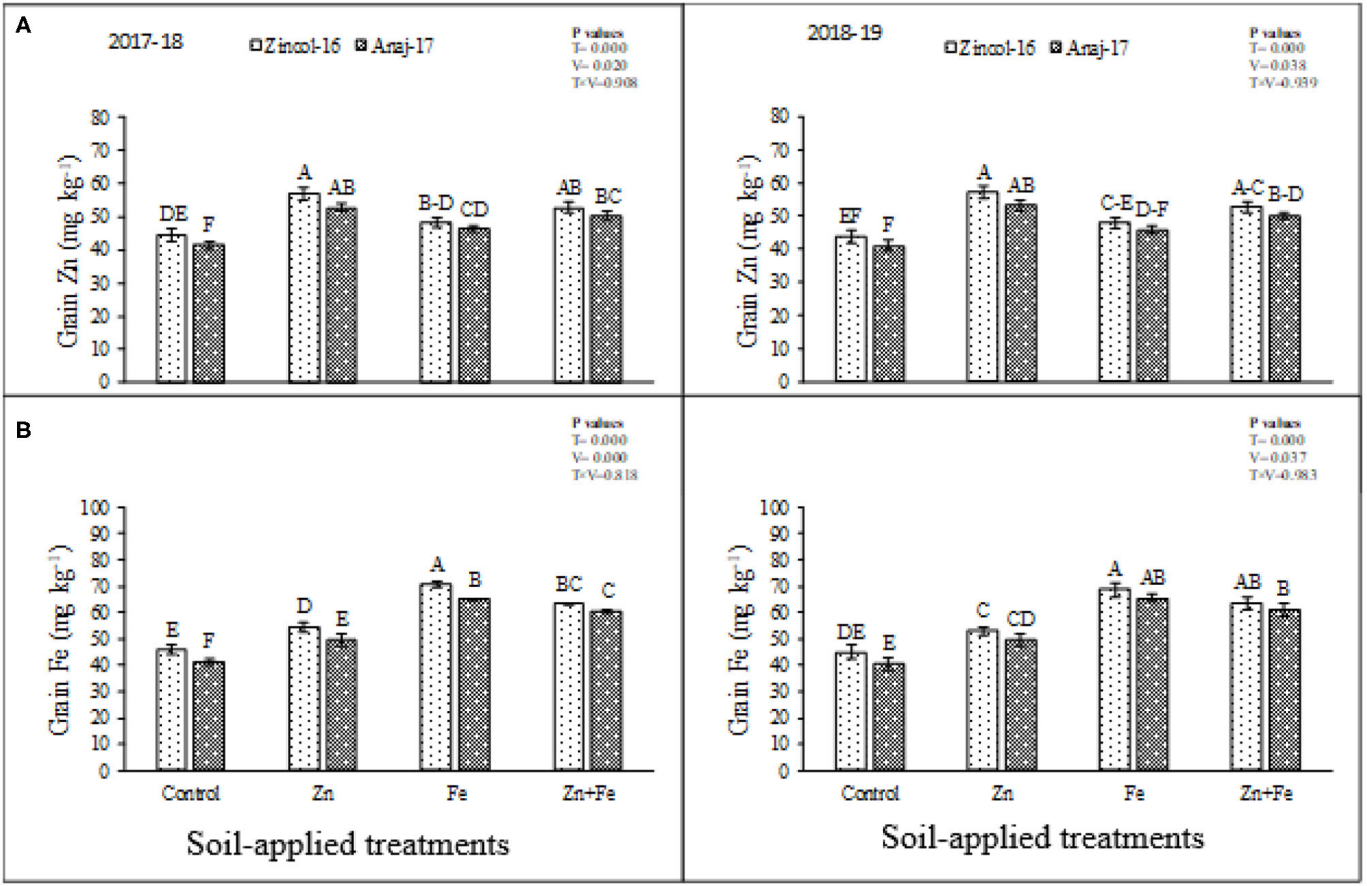
Impact of soil-applied Zn and Fe on Zn **(A)**, and Fe **(B)** contents in grains of wheat cultivars grown during 2017–2018 and 2018–2019 growing seasons. Means sharing the same letter did not differ significantly at *p* > 0.05. Error bars depict the standard error of means.

**Figure 3:**
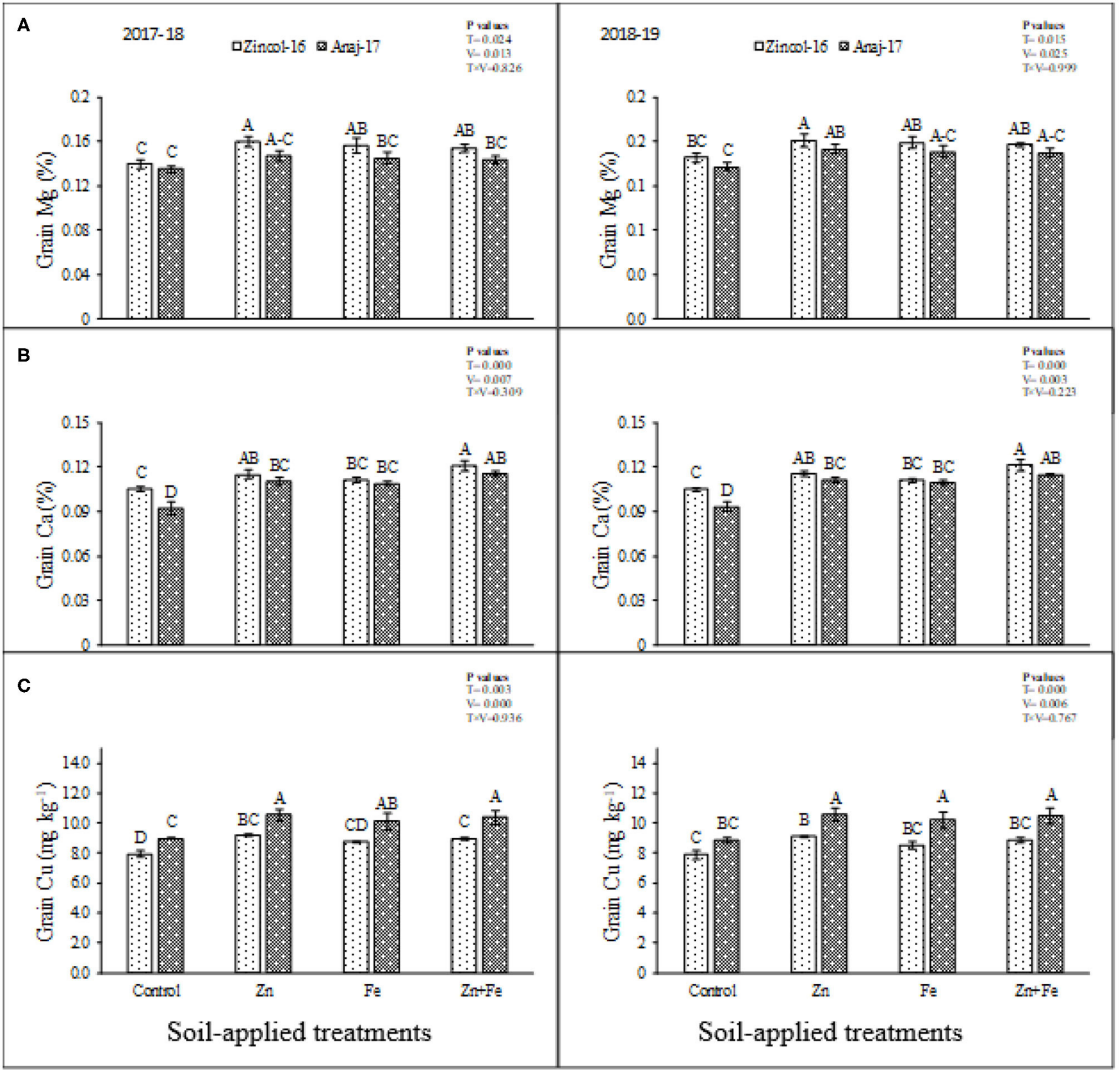
Impact of soil-applied Zn and Fe on Mg **(A)**, Ca **(B)**, and Cu **(C)** contents in grains of wheat cultivars grown during 2017–2018 and 2018–2019 growing seasons. Means sharing the same letter did not differ significantly at *p* >0.05. Error bars depict the standard error of means.

There were significant differences (*p* ≤ 0.05) in Zn and Fe accumulation in grains of cultivars and soil-applied treatments (Figure 2). The interactive effect of fertilizers and cultivars showed no significant difference for the planting season of 2017 and 2018. The sole application of Zn produced maximum Zn contents in the grains while minimum in control for both cultivars (Figure 2A). Cultivar Zincol-16 was found more efficient in Zn accumulation as compared to Anaj-17 (Figure 2) during both experimental years. Fe accumulation was significantly (*p* ≤ 0.05) improved by soil-applied treatments. Maximum improvement was recorded by sole application of Fe, while minimum in control (Figure 2B). Zincol-16 accumulated higher content of Fe in grains as compared to Anaj-17 during both experimental years. Based on the findings of the study, significant (*p* ≤ 0.05) improvement was recorded regarding the concentrations of Mg, Ca, and Cu in the grains of wheat cultivars by the soil-applied treatments (Figure 3). All the treatments enhanced the concentration of Mg in grains as compared to control (Figure 3A). Combined application produced the highest concentration of Ca in grains, which were statistically at par with sole Zn application while minimum in control (Figure 3B). Zincol-16 accumulated higher concentrations of Mg and Ca in their grains as compared to the Anaj-17 cultivar during the first and second years of experimentation. All the soil-applied treatments significantly (*p* ≤ 0.05) increased Cu concentration in the wheat grains as compared to control (Figure 3C). Sole application of Zn produced maximum Cu contents in grains, which were statistically at par with a combined application, and sole application of Fe during 2017–2018.

### Starch, Wet Gluten, and Protein Contents

**Figure 4:**
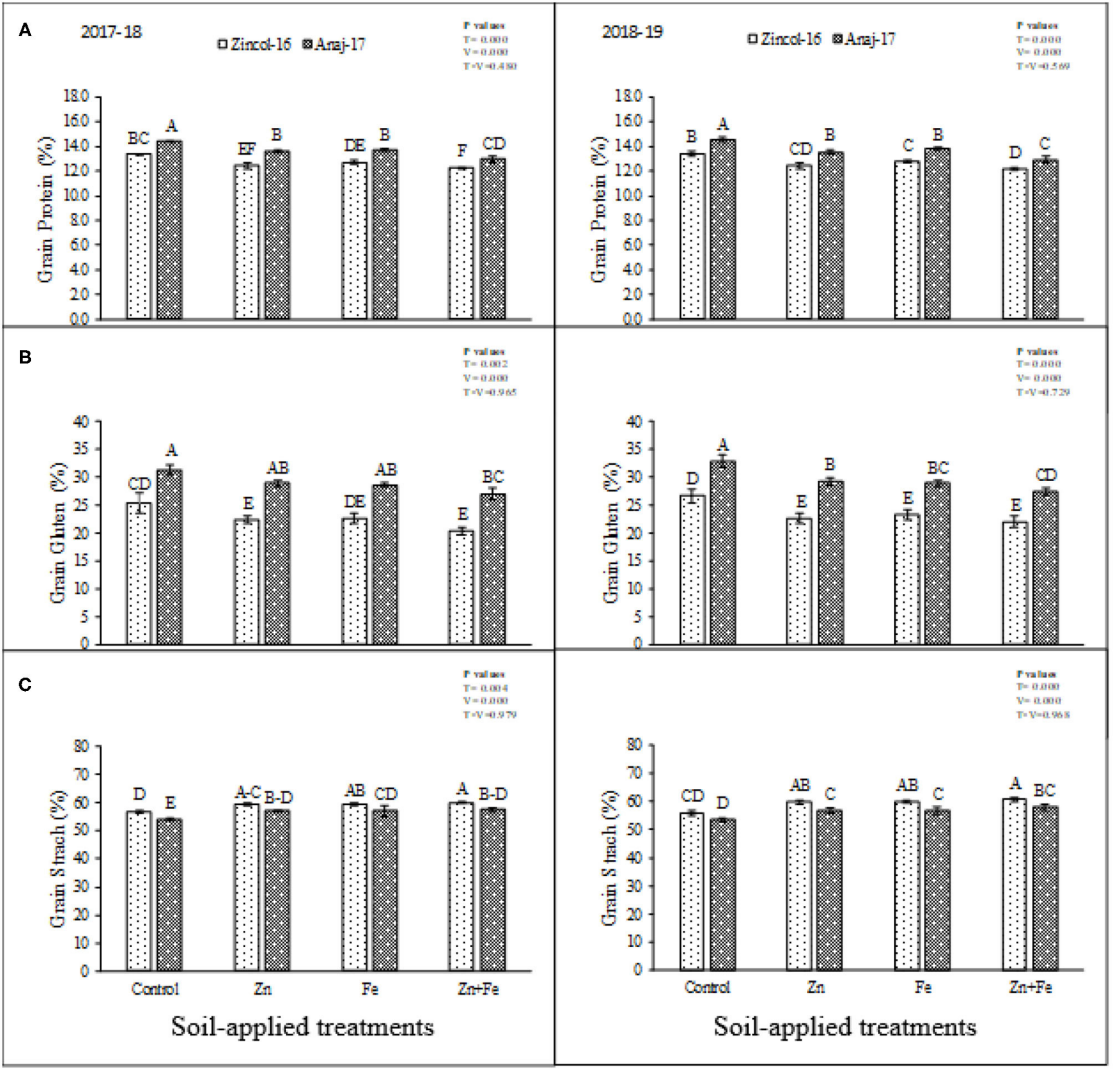
Impact of soil-applied Zn and Fe on grain protein **(A)**, grain gluten **(B)**, and grain starch **(C)** of wheat cultivars grown during 2017-2018 and 2018-2019 growing seasons. Means sharing the same letter did not differ significantly at *p* > 0.05. Error bars depict the standard error of means.

There were significant differences (*p* ≤ 0.05) in starch, wet gluten, and proteins concentration among the soil-applied treatments and between the wheat cultivars in the first as well as the second year of experimentation, while their interactive effect was found nonsignificant. Cultivar Anaj-17 synthesized more protein contents under control treatment (Figure 4A). Combined application produced the lowest protein contents in the Zincol-16 cultivar. Overall, Anaj-17 performed better regarding protein contents in grains as compared to Zincol-16 in both years of study. Maximum gluten contents were recorded in control, while minimum by combined application of Zn and Fe, which were statistically at par with a sole application of either Zn or Fe (Figure 4B). Cultivar Zincol-16 synthesized more gluten contents comparatively than Anaj-16 during 2017 and 2018. Sole application of Fe produced maximum starch concentration in grains while minimum in control. Maximum starch concentration was recorded in Zincol-16, while Anaj-17 accumulated low-starch concentration in their grains. The combined application of Zn and Fe produced more starch contents than the sole application of Zn, while less than the sole application of Fe during both experimental years (Figure 4C).

### Growth and Yield Parameters

Soil-applied Zn and Fe significantly (*p* ≤ 0.05) improved the growth and yield parameters of both wheat cultivars in both seasons of wheat cultivation. The combined application of Zn and Fe at 10 and 12 kg ha^−1^ produced a maximum number of tillers (m^−2^) (Table 2), while the minimum was found in the control treatment. Sole application of Zn and Fe produced 365 and 361 numbers of tillers, respectively, which were statistically at par with each other in the first year of study. Anaj-17 produced a greater number of tillers as compared to Zincol-16 in both years of experimentation. A similar trend was also found in the plant height of both cultivars in response to soil-applied treatments (Table 2). Spike length was increased significantly (*p* ≤ 0.05) by the soil-applied treatments, and significant variation was also found within the cultivars (Table 2). Combined application produced the highest number of spikelets per spike, grains per spike, and 1,000-grain weight (Table 3) as compared to sole application of either Zn or Fe, while minimum mean values were found in control throughout the course of theexperimentation.

**Table 2:** Impact of soil-applied zinc (ZnSO_4_) at 10 kg ha^−1^ and iron (FeSO_4_) at 12 kg ha^−1^ on a number of tillers, plant height, and spike length of wheat varieties cultivated during 2017-2018 and 2018-2019 growing seasons (*n* = 3).

**Treatments**	**Number of tillers (m** ^**−2**^ **)**						**Plant height (cm)**						**Spike length (cm)**					
	**2017–2018**			**2018–2019**			**2017–2018**			**2018–2019**			**2017–2018**			**2018–2019**		
	**Zincol-16**	**Anaj-17**	**Mean (T)**	**Zincol-16**	**Anaj-17**	**Mean (T)**	**Zincol-16**	**Anaj-17**	**Mean (T)**	**Zincol-16**	**Anaj-17**	**Mean (T)**	**Zincol-16**	**Anaj-17**	**Mean (T)**	**Zincol-16**	**Anaj-17**	**Mean (T)**
Control	339	350	344C	331	360	345C	101.9	98.8	100.3C	101.6	99	100.3C	9.5	9.7	9.6C	9.6	9.9	9.7C
Sole Zn	358	372	365B	362	379	371B	106	103.3	104.6B	105.7	103.2	104.4B	10.1	10.6	10.3AB	10.1	10.7	10.4AB
Sole Fe	354	368	361B	359	371	365B	104.7	103	103.8B	104.7	102.7	103.7B	9.9	10.3	10.1 B	10	10.4	10.2B
Zn + Fe	367	381	374A	369	387	378A	107.9	105.3	106.6A	107.2	105.1	106.2A	10.4	10.9	10.7 A	10.5	10.8	10.6A
Mean (C)	355 B	368A		355B	374A		105.1A	102.6B		104.8A	102.5B		10.0 B	10.4A		10.0B	10.5A	
**LSD**	*T =* 4.79; *C =* 3.39; T × *C =* ns			*T =* 5.65; *C =* 4.0; T × *C =* ns			*T =* 1.80; *C =* 1.27; T × *C =* ns			*T =* 1.74; *C =* 1.23; T × *C =* ns			*T =* 0.36; *C =* 0.25; T × *C =* ns			*T =* 0.27; *C =* 0.19; T × *C =* ns		

*Different letters within the same column indicate statistically significant differences at p ≤ 0.05, T, treatment; C, cultivar; T × C, interaction; ns, nonsignificant*.

**Table 3:** Impact of soil-applied zinc (ZnSO_4_) at 10 kg ha^−1^ and iron (FeSO_4_) at 12 kg ha^−1^ on number of spikelets per spike, number of grains per spike, and 1,000-grain weight of wheat varieties cultivated during 2017–2018 and 2018–2019 growing seasons (*n* = 3).

**Treatments**	**Number of spikelet's per spike**						**Number of grains per spike**						**1,000-grain weight (g)**					
	**2017–2018**			**2018–2019**			**2017–2018**			**2018–2019**			**2017–2018**			**2018–2019**		
	**Zincol-16**	**Anaj-17**	**Mean (T)**	**Zincol-16**	**Anaj-17**	**Mean (T)**	**Zincol-16**	**Anaj-17**	**Mean (T)**	**Zincol-16**	**Anaj-17**	**Mean (T)**	**Zincol-16**	**Anaj-17**	**Mean (T)**	**Zincol-16**	**Anaj-17**	**Mean (T)**
Control	16	18	17C	17	18	18C	41	44	43C	42	44	43C	32.4	34.7	33.5D	32.7	34.9	33.8D
Sole Zn	18	19	18B	18	19	19B	47	49	48B	48	49	48AB	36.8	37.8	37.3B	37.1	38.2	37.7B
Sole Fe	18	19	18B	18	19	19B	46	48	47B	47	47	47B	35.5	36.2	35.9C	35.8	36.4	36.1C
Zn + Fe	19	20	20A	19	20	20A	49	51	50A	49	51	50A	38.1	39.2	38.6A	38.4	39.7	39.0A
Mean (C)	18B	19A		18B	19A		46B	48A		46B	48A		35.7B	37.0A		36.0B	37.3A	
**LSD**	*T =* 0.65; *C =* 0.46; T × *C =* ns			*T =* 0.68; *C =* 0.48; T × *C =* ns			*T =* 1.27; *C =* 0.89; T × *C =* ns			*T =* 1.82; *C =* 1.29; T × *C =* ns			*T =* 1.03; C = 0.73; T × *C =* ns			*T =* 1.10; C = 0.78; T × *C =* ns		

*Different letters within the same column indicate statistically significant differences at p ≤ 0.05, T, treatment; C, cultivar; T × C, interaction; ns, nonsignificant*.

Cultivar Anaj-17 performed better and produced more biological yield, grain yield, and harvest index as compared to Zincol-16, which produced low biological yield, grain yield, and harvest index (Table 4). The combined application of Zn and Fe produced maximum biological yield as compared to other treatments. Sole application of Zn and Fe produced less biomass as compared to combined application, while minimum biomass was produced in control. The highest grain yield was produced by the combined application while the lowest was in control. The sole application of Zn comparatively produced more grain yield than the sole application of Fe. The maximum harvest index was recorded by the combined application while the minimum in control treatment during the first and second years of the experimentation (Table 4).

**Table 4:** Impact of soil-applied zinc (ZnSO_4_) at 10 kg ha^−1^ and iron (FeSO_4_) at 12 kg ha^−1^ on biological yield, grain yield, and harvest index of wheat varieties cultivated during 2017–2018 and 2018–2019 growing seasons (*n* = 3).

**Treatments**	**Biological yield (t ha** ^**−1**^ **)**						**Grain yield (t ha** ^**−1**^ **)**						**Harvest index (%)**					
	**2017–2018**			**2018–2019**			**2017–2018**			**2018–2019**			**2017–2018**			**2018–2019**		
	**Zincol-16**	**Anaj-17**	**Mean (T)**	**Zincol-16**	**Anaj-17**	**Mean (T)**	**Zincol-16**	**Anaj-17**	**Mean (T)**	**Zincol-16**	**Anaj-17**	**Mean (T) (T)**	**Zincol-16**	**Anaj-17**	**Mean (T)**	**Zincol-16**	**Anaj-17**	**Mean (T)**
Control	11	11.1	11.1C	11.2	11.5	11.3C	3.2	3.59	3.39D	3.27	3.62	3.45D	28.8	32.7	30.7D	29.4	31.6	30.5C
Sole Zn	11.9	12.1	12.0B	12.2	12.5	12.3AB	3.74	4.12	3.93B	3.8	4.17	3.98B	30.9	34.7	32.8B	31.4	33.4	32.4AB
Sole Fe	11.7	12	11.9B	12	12.4	12.2B	3.61	3.93	3.77C	3.67	4.01	3.84C	30.2	33.5	31.8C	30.7	32.3	31.5BC
Zn + Fe	12.1	12.4	12.3A	12.4	12.7	12.5A	3.98	4.29	4.13A	4.03	4.35	4.19A	32.2	35.4	33.8A	32.6	34.3	33.5A
Mean (C)	11.7B	11.9A		11.9B	12.3A		3.63B	3.98A		3.69B	4.04A		30.5B	34.1A		31.0B	32.9A	
**LSD**	*T =* 0.13; *C =* 0.09; T × *C =* ns			*T =* 0.13; *C =* 0.09; T × *C =* ns			*T =* 0.10; *C =* 0.07; T × *C =* ns			*T =* 0.07; *C =* 0.14; T × *C =* ns			*T =* 0.78; *C =* 0.55; T × *C =* ns			*T =* 1.19; *C =* 0.84; T × *C =* ns		

*Different letters within the same column indicate statistically significant differences at p ≤ 0.05, T, treatment; C, cultivar; T × C, interaction; ns, nonsignificant*.

### Correlation

**Figure 5:**
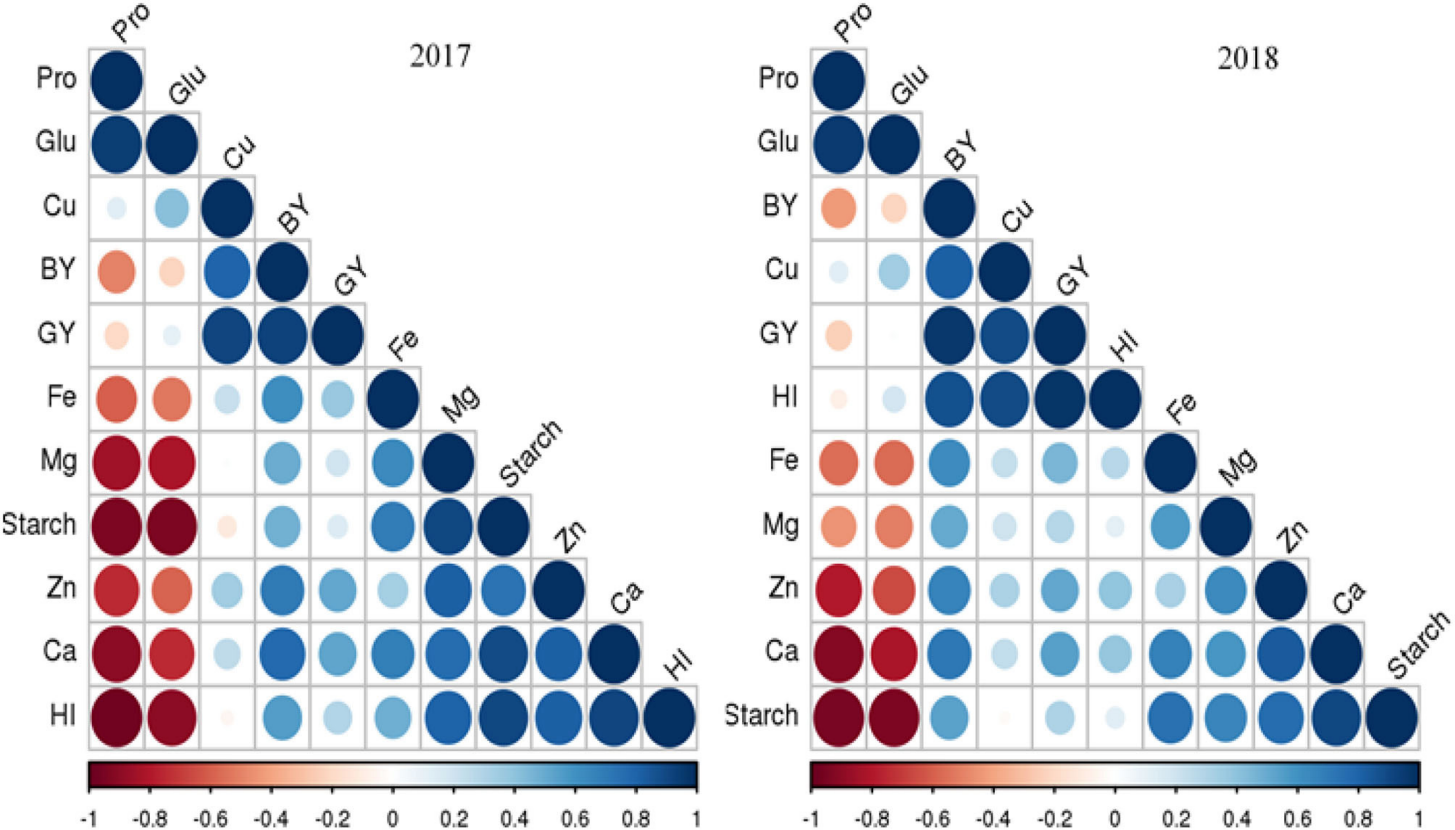
Pearson correlations among different yield attributes and grain quality traits of wheat cultivars grown under soil-applied Zn and Fe.

Pearson correlation revealed a strong linear relationship among protein and gluten, while the negative correlation with harvest index, grain yield, biological yield, starch, protein, Ca, Mg, Fe, and Zn, while Cu showed a positive correlation with protein and gluten content during the 2017 experimental year. During 2018, biological yield, Cu, grain yield, harvest plus, Fe, Mg, Zn, Ca, and starch showed a linear relationship among them. Similarly, protein and gluten depicted a strong linear relationship among them, while protein and gluten showed a negative correlation with all response variables as shown in Figure 5.

## Discussion

This field study explored the effects of soil-applied zinc sulfate and iron sulfate fertilization on crop yield and grain Zn and Fe contents in wheat cultivars, Zincol-16 (Zn enriched) and Anaj-17 (Zn deficient). Micronutrient-deficient soils are increasing due to the frequent growth of exhaustive crops and intensive use of fertilizer, i.e., nitrogen, potassium, and phosphorus (20). The soils having <0.5 mg kg^−1^ DTPA extractable Zn are usually considered as potentially Zn deficient and could be responsive to soil Zn fertilization (44). Rengel (45) stated that calcareous soils are Fe deficient, having high pH. Zn and Fe deficiency is more common in Pakistani soil that has high pH, free CaCO_3_, and HCO^−3^, which inhibit the accessibility of Fe and Zn to the plants (16, 19). Based on these criteria, current study soils seemed to be potentially Zn and Fe deficient. Therefore, there was an expectation for enhanced ions contents in grain and yield with soil fertilization of zinc sulfate and iron sulfate.

Mineral accumulation in grains is an imperative indicator for evaluating the capacity of plants to take up the beneficial elements that reveal the biofortification potential of plants. Zn accumulation in grains of cultivars by soil applied treatments was significantly improved, and cultivar Zincol-16 was found more efficient in Zn accumulation as compared to Anaj-17 (Figure 2A). Iron (Fe) accumulation also was significantly improved by soil-applied treatments. Maximum improvement was recorded by sole application of Fe at 12 kg ha^−1^. In the case of cultivars, Zincol-16 accumulated higher content of Fe in grains as compared to Anaj-17 (Figure 2B). Our results were in line with Zou et al. (39); they stated that soil application of Zn increased Zn contents in wheat grains. The present study also was supported by Zulfiqar et al. (1), who reported Fe soil application increased Fe contents in wheat grains. These results were supported by Ramzan et al. (5), who reported Zn and Fe soil application increased Cu, Mg, and Ca contents in wheat grains. Imtiaz et al. (46) stated that Zn foliage applied had an adverse effect on Cu contents in wheat grains.

Zn and Fe contribute to photosynthesis, chlorophyll formation, metabolism of starch formation, and enzyme carbonic anhydrase, accelerating carbohydrate formation. The maximum concentrations of Zn and Fe are necessary to accumulate suitable carbohydrate contents (47, 48). There were significant differences in starch concentration among the soil-applied treatments and between the wheat cultivars. Our outcomes were supported by Kinaci and Kinaci (49), who reported that Zn soil supplementation significantly increased the starch in barley. Mousavi et al. (50) also reported that Zn soil applied markedly enhanced the starch in the potato. Our outcomes were in contrast with Keram et al. (51), who reported that soil-applied Zn significantly increased wet gluten. Protein contents were also significantly reduced by soil-applied treatments. Ramzan et al. (5) noted that soil-applied Zn and Fe significantly decreased the protein content in spring wheat. Mugenzi et al. (52) reported that Fe and Zn application, either sole or combined, showed a nonsignificant effect on protein content.

The combined application significantly produced maximum growth and yield attributes in wheat cultivars. A number of tillers have key importance in achieving the final yield in a wheat crop. The combined application of Zn and Fe at 10 and 12 kg ha^−1^ produced a maximum number of tillers per unit area. Anajs-17 produced a greater number of tillers as compared to Zincol-16 (Table 2). Our findings are also supported by Jalal et al. (53), who stated that the combined application of Zn and Fe significantly enhanced the number of tillers per unit area. They also reported that a number of productive tillers are very important in determining the yield in cereal crops. Outcomes of the present experimentation are also in line with Boorboori et al. (54), who stated that application of Zn and Fe through soil incorporation has a positive influence on tillers, and more tillers were attained by the soil-applied Zn and Fe. Plant height is one of the main parameters that determine the final yield of a crop. Plant height is a function of the combined effect of both genetic and environmental factors. Significant differences were observed in plant height as a result of combined application of Zn and Fe. Combined application produced the highest plant and spike length (Table 2) in wheat plants. More height of plants might be due to the involvement of Zn and Fe in cell division, cell expansion, activities of meristematic tissues, and photosynthetic activities.

In the current experimentation, the combined application of Zn and Fe is responsible for a maximum number of grains per spike, 1,000-grain weight, and spikelets per spike (Table 3). Zayed et al. (55) reported similar findings that 1,000-kernel weight in rice was significantly enhanced by the mixed application of Zn and Fe as compared to sole application. Bameri et al. (56) concluded from their experimentation that the application of Zn significantly enhanced the grain numbers per spike, productive tillers, 1,000-grain weight, and spike length. Hassan et al. (57) found the positive impact of Zn on the growth parameters of the wheat crop. They stated that the application of Zn improved the Zn dietary standards, more than 1,000-grain weight, the maximum number of grains per spike, as well as spikelets. This, perchance, is because of the fact that Zn is an important element and shows a key role in regulating the auxin concentration throughout the plant body, biosynthesis of indole acetic acid. Zn also controls the physiological and biochemical processes and stimuli for the initiation of primordia regarding reproductive growth. It has a positive influence on the translocation of required metabolites from the source to the sink of plants. In the case of Fe, it is the component of the photosynthetic apparatus as well as its rate and formation of chlorophyll. The application of Fe significantly improved the yield and its contributing factors. It is also reported that enhanced photosynthesis and respiration rates, more crop growth, and improved physiological and biochemical processes were observed by the application of Fe, Zn, and Mn (58). Rehman et al. (31) and Farooq et al. (59) also reported that the application of minerals either alone or in combination with growth promoters improved the growth attributes of crops. Tabaxi et al. (60) stated that the application of various fertilizers or mineral elements improved the agronomic and quality characteristics of crops.

Maximum grain and biological yield, and harvest index were observed by the combined application of Zn and Fe in the current study (Table 4). This improvement in the biological and economical yield is due to the fact that zinc has a catalytic and constructive role in the physiological and biochemical activities and in respiration and photosynthesis processes and thus resulting in higher economical yield. Zain et al. (61) concluded that supplementation of nutrients, particularly microelements, is responsible for improved harvest index, biological and grain yields linked with more tillers, number of grains per spike, and 1,000-grain weight. The application of inorganic fertilizers and mineral elements is considered a helpful practice in maintaining crop productivity with improved soil fertility to achieve maximum plant growth and economical yield under stressful conditions (62, 63). The conversion of nitrates to ammonia is also triggered by the Zn that ultimately improves the economical out of a wheat crop. In the present experimentation, the grain yield was also improved by the application of Fe at 10 kg ha^−1^ because Fe is useful for translocation of assimilates and photosynthates from the source toward the sink, particularly grains in the case of wheat. Moreover, Fe is also essential for optimum rates of respiration and photosynthesis that results in maximum accumulation of biomass. Harvest index (HI) is a sign regarding the translocation and partitioning of photosynthates and dry matter toward reproductive structure like grains in the case of a wheat crop. The findings of the current experimentation are supported by previous studies that the application of various doses of Zn and Fe significantly improved the harvest index of wheat (53) and maize crops (64).

## Conclusion

Cultivar Zincol-16 produced maximum ions concentration, starch contents, and wet gluten as compared to Anaj-17. Yield and growth attributes, especially the number of tillers, plant height, number of grains, and 1,000-grain weight, were also significantly improved by the combined application of Zn and Fe as compared to the sole application of Zn or Fe. The combined application of Zn and Fe produced the highest biological and grain yield. Cultivar Anaj-17 was found more responsive regarding growth and yield attributes comparatively. Findings of the present experimentation explored that combined application of Zn and Fe at 10 and 12 kg ha^−1^, respectively, produced good-quality grains with a maximum productivity of bread wheat cultivars grown under calcareous soil.

## Data Availability Statement

The original contributions presented in the study are included in the article/supplementary material, further inquiries can be directed to the corresponding author/s.

## Author Contributions

MBH, YR, and SK proposed the idea and funding was secured by Z-HD, AA-H, and AA. The experiments were conducted by MBH, MN, SR, HS, JA, and MH, as well as data collection. The first draft was prepared by SK, MBH, DI, SB, NZ, NR, and SI. The draft was finalized after careful review by JA, SB, SK, and MH. SK and Z-HD submitted the final draft as corresponding authors. All authors contributed to the article and approved the submitted version.

## Funding

This work was supported by the Key Realm R&D Program of Guangdong Province (Nos. 2020B1111350002 and 2020B0202080002), the special project in key areas of Guangdong Province Ordinary Universities (No. 2020ZDZX1003), the Guangdong Provincial Special Fund for Modern Agriculture Industry Technology Innovation Teams (No. 2019KJ140), and the National Natural Science Foundation of China (No. 21407155).

## Conflict of Interest

The authors declare that the research was conducted in the absence of any commercial or financial relationships that could be construed as a potential conflict of interest.

## Publisher's Note

All claims expressed in this article are solely those of the authors and do not necessarily represent those of their affiliated organizations, or those of the publisher, the editors and the reviewers. Any product that may be evaluated in this article, or claim that may be made by its manufacturer, is not guaranteed or endorsed by the publisher.
